# Spatiotemporal variation in ecophysiological traits align with high resolution niche modelling in the short-range banded ironstone endemic *Aluta quadrata*

**DOI:** 10.1093/conphys/coae030

**Published:** 2024-05-24

**Authors:** Wolfgang Lewandrowski, Emily P Tudor, Hayden Ajduk, Sean Tomlinson, Jason C Stevens

**Affiliations:** Kings Park Science, Department of Biodiversity, Conservation and Attractions, 2 Kattidj Close, Kings Park, WA 6005, Australia; School of Biological Sciences, University of Western Australia, Nedlands, WA 6009, Australia; Kings Park Science, Department of Biodiversity, Conservation and Attractions, 2 Kattidj Close, Kings Park, WA 6005, Australia; School of Biological Sciences, University of Western Australia, Nedlands, WA 6009, Australia; Rio Tinto, Central Park, 152–158 St Georges Terrace, Perth, Western Australia 6000, Australia; Kings Park Science, Department of Biodiversity, Conservation and Attractions, 2 Kattidj Close, Kings Park, WA 6005, Australia; Geospatial Science, Department of Biodiversity, Conservation and Attractions, Kensington, WA 6151, Australia; School of Biological Sciences, University of Adelaide, Adelaide, SA 5000, Australia; Kings Park Science, Department of Biodiversity, Conservation and Attractions, 2 Kattidj Close, Kings Park, WA 6005, Australia

**Keywords:** Chlorophyll fluorescence, conservation biology, drought, ecophysiology, edaphic factors, gas exchange, rare species, restoration, soil microclimate, water potential

## Abstract

Defining plant ecophysiological responses across natural distributions enables a greater understanding of the niche that plants occupy. Much of the foundational knowledge of species’ ecology and responses to environmental change across their distribution is often lacking, particularly for rare and threatened species, exacerbating management and conservation challenges. Combining high-resolution species distribution models (SDMs) with ecophysiological monitoring characterized the spatiotemporal variation in both plant traits and their interactions with their surrounding environment for the range-restricted *Aluta quadrata* Rye & Trudgen, and a common, co-occurring generalist, *Eremophila latrobei* subsp. *glabra* (L.S.Sm.) Chinnock., from the semi-arid Pilbara and Gascoyne region in northwest Western Australia. The plants reflected differences in gas exchange, plant health and plant water relations at sites with contrasting suitability from the SDM, with higher performance measured in the SDM-predicted high-suitability site. Seasonal differences demonstrated the highest variation across ecophysiological traits in both species, with higher performance in the austral wet season across all levels of habitat suitability. The results of this study allow us to effectively describe how plant performance in *A. quadrata* is distributed across the landscape in contrast to a common, widespread co-occurring species and demonstrate a level of confidence in the habitat suitability modelling derived from the SDM in predicting plant function determined through intensive ecophysiology monitoring programmes. In addition, the findings also provide a baseline approach for future conservation actions, as well as to explore the mechanisms underpinning the short-range endemism arid zone systems.

## Teaser

We used species distribution modelling to estimate habitat suitability for the short range endemic, *Aluta quadrata*, in the arid northwest of Australia and conducted ecophysiological monitoring across different seasons. We found higher plant performance in the high-suitability site, providing evidence to support the species distribution model for *A. quadrata*.

## Introduction

Plant species demonstrate considerable variation in their geographic range and distribution, and their capacity to respond to environmental stressors has become critical underpinnings for management initiatives (Madliger *et al.,* 2018; Maxwell *et al.,* 2019). Identifying management initiatives to support conservation actions is critical, particularly when plant populations are exposed to increasing disturbance pressure (Felton and Smith, 2017). For short-range endemics (SRE, species with narrow distributions; Lavergne *et al.,* 2004), knowledge of the factors shaping their distributions and capacity to cope with environmental pressures is often lacking (Bartholomew *et al.,* 2022). This is often a significant challenge for management initiatives, as SREs commonly have tenuous population numbers, localized to specialized habitat characteristics (Gosper *et al.*, 2020; Howard *et al.,* 2020). Consequently, many threatened SREs are inferred to be highly vulnerable to changes in their environment (Bartholomeus *et al.,* 2011). Therefore, monitoring their persistence, as well as mitigating local impacts on communities, is critical to counter potential population losses, range contractions, and ultimately extinctions (Maxwell *et al.,* 2019; Watson *et al.,* 2020; Cazzolla Gatti *et al.,* 2022).

Many edaphic and climatic factors, including soil physical structure, landscape topography, extreme temperatures and unpredictable rainfall, as well as unprecedented disturbance events (e.g. cyclone activity, floods, fires), are associated with shaping species distribution patterns (Maxwell *et al.,* 2019; Pascual *et al.,* 2022). Species distribution models (SDMs) are an effective tool in spatial ecology, environmental management and conservation for describing, explaining and predicting species’ likely biogeography, particularly in response to threatening processes such as climate change or environmental degradation (Casazza *et al.,* 2021). Correlative or phenomenological models are the most common approaches (Elith and Leathwick, 2009), identifying statistical relationships between species occurrence and local environmental factors to characterize and relate landscape elements that are critical to the distributions of species (Kearney and Porter, 2009). The resulting predictions of the probability of occurrence across a landscape are often used to infer habitat suitability (Gogol-Prokurat, 2011; Guisan *et al.,* 2017) However, the challenge with SREs is that there are often discrepancies between the resolution of the spatial data and the geographical range of the occurrence data of the species in focus (Tomlinson *et al.,* 2020). This can often lead to predicting large areas in the landscape that define high probability of occurrence despite known absences that may be due to geographic or dispersal barriers (Byrne, 2019; Casazza *et al.,* 2021). Additionally, SREs are often associated with higher degrees of specialization or constraints to specific environmental conditions in an otherwise very challenging or stochastic landscape (Lavergne *et al.,* 2004; Lannuzel *et al.,* 2021). As such, edaphic and topographic data may be more appropriate for modelling SREs, as models can be constructed at a resolution that is biologically meaningful and informative across highly localized distributions (Tomlinson *et al.,* 2020). Whilst correlative modelling approaches describe the patterns of association between species occurrence and environmental or climatic data, they often fall short in delivering causal explanations for the projected outcome (Peterson *et al.,* 2015). In addition, correlative models are limited in their transferability to novel or changing environments and there are calls for *in situ* model validation and interpretations of causation (Kearney and Porter, 2009).

In conservation and restoration contexts, there are increasing demands for using ecophysiological measures to help measure performance, sensitivities and resilience of plants in response to natural as well as manipulated environments (Cooke *et al.,* 2021; Schönbeck *et al.,* 2023). Ecophysiological surveys can provide critical insights into the patterns and processes governing persistence, especially when responses demonstrate spatiotemporal variation (Grossman, 2023) and can be used as a tool to validate correlative, occurrence-based SDMs by quantifying plant–environmental responses (Tomlinson *et al.,* 2021). This is because plant responses to the environment vary significantly, both spatially and temporally, with periods of ecophysiological activity and inactivity driven by seasonal moisture and temperature patterns (Schwinning and Sala, 2004; Hamerlynck and Huxman, 2009). Measures of gas exchange, chlorophyll fluorescence and leaf water potentials provide important ecophysiological indicators of plant performance in terms of physiological function, health and water stress (Madliger *et al.,* 2018; Valliere *et al.,* 2021; Schönbeck *et al.,* 2023). In dryland ecosystems, increased physiological activity is typically triggered by sustained rainfall leading to elevated gas exchange, plant water use and productivity (Manzoni *et al.,* 2014; Tarin *et al.,* 2020), which can lead to improved plant health and reproductive success (Huxman *et al.,* 2004). By contrast, during drought or thermal stress, plants undertake morphological and physiological adjustments, including leaf senescence, modified gas exchange, water use and nutrient uptake (Chaves *et al.,* 2003). Prolonged periods of inactivity may risk reductions in cellular repair, and ultimately mortality (Larcher, 2003). These processes may become exacerbated depending on the environmental factors shaping the local niche, and plants at the edge of their distribution may experience greater environmental stress due to unfavourable niche characteristics (Abeli *et al.,* 2014). Therefore, understanding ecophysiological responses of where species may grow and persist or where they may perish can help achieve targeted management actions to aid conservation more broadly.

Here, we constructed a high-resolution SDM informed by edaphic and topographic spatial data to identify the factors associated with the distribution of a narrow-range banded ironstone endemic *Aluta quadrata* Rye & Trudgen in arid tropical northwestern Australia. Using a suite of ecophysiological measures to quantify seasonal variation in plant performance in contrasting sites, this study develops an understanding of the niche that this plant occupies in contrast to a common co-occurring *Eremophila latrobei* F.Muell., which has a widespread distribution throughout dryland ecosystems in Australia. As such, the broad research objectives of the study were to: 1) characterize the niche that *A. quadrata* occupies and establish meaningful biological correlates between modelled probability of occurrence and plant performance, 2) define the ecophysiological interactions of *A. quadrata* in sites of contrasting probabilities and validate whether modelled probability corresponds to differential physiological performance and 3) evaluate the differences in ecophysiological performance of the SRE *A. quadrata* and the widespread generalist, *Eremophila latrobei* subsp. *glabra* (from hereon referred to as *E. latrobei*). We expected that there would be associations between ecophysiological performance and the SDM output for *A. quadrata*, with individuals in high-suitability locations presenting elevated physiological performance compared to individuals in low-suitability locations. Moreover, we expected that the generalist, *E. latrobei,* would demonstrate different spatiotemporal patterns in ecophysiological functioning to *A. quadrata*, but whether these would imply higher or lower performance should vary depending on biogeography.

## Materials and Methods

### Study location and species

The study area is located in the southern Pilbara (PIL) and northern Gascoyne (GAS) region, in the northwest of Western Australia. The climate in this area is typically characterized as semi-arid/arid, with >70% of the annual rainfall occurring during the hot summer period (average maximum air temperature: 38–41°C; December–March; Charles *et al.,* 2013, Bureau of Meteorology, 2023). Autumn, winter and spring seasons are typically characterized by dry, warm days and cool nights (average maximum air temperatures: 25–35°C; Bureau of Meteorology, 2023), with infrequent or little rainfall. The landscape is characterized by elevated ranges, ridges, mesa outcrops of the Hamersley Ranges in the south and Chichester Ranges in the north (Pepper *et al.,* 2013). An extensive network of rivers, drainage flats and floodplain systems of the Fortescue Marsh and the GAS region to the south envelope the Hamersley Ranges and the coastal Roeburne Plains to the north of the Chichester Ranges (Pepper *et al.,* 2013). The vegetation is dominated by *Triodia* hummock grasslands on rocky skeletal soils, with *Acacia* and *Grevillea* mosaic shrub lands and mallees and trees along deeper soils and along riparian river and creek systems (McKenzie *et al.*, 2009).

*Aluta quadrata* is a medium-sized shrub, ~0.8–2.6 m in height, with white flowers and smooth, grey or pale brown fissured bark, and yellow-green needle-like foliage (Western Australian Herbarium, 1998-). Plant populations are restricted to a single banded ironstone range on the southern edge of the Hamersley Range in the PIL region, northwest Western Australia (Byrne *et al.,* 2017; Binks *et al.,* 2019) and grow in steep rocky slopes, gorges and gullies, with a preference for southern-facing slopes of rugged topography in skeletal soils, including Brockman Iron Formation substrates (Byrne *et al.,* 2017). Currently there are an estimated 41 136 individuals distributed across three geographically discrete populations (Western Ranges, Pirraburdoo and Channar; Supplementary Fig. S1). We made ecophysiological comparisons between *A. quadrata* and a widespread common co-occurring plant species, *E. latrobei*, a medium-sized shrub, ~0.3–3 m in height, with red- or pink-coloured flowers, and grey- to green-coloured leaves (Western Australian Herbarium, 1998-). *Eremophila latrobei* plants are widely distributed throughout the arid zone region of the continent, sharing similar habitat preferences with *A. quadrata*, growing in stoney red sandy soils on ironstone hills, and more broadly across sandy soils on plains. Like *A. quadrata, E. latrobei* shares similar plant functional traits, with flowering occurring following summer rainfall between April and October, producing woody fruit, as well as becoming senescent plants by shedding leaves as seasons transition into the dry (Richmond and Chinnock, 1994; Brown and Buirchell, 2011).

### Species distribution modelling

We constructed a species distribution model for *A. quadrata* using presence point data and publicly available datasets describing the physical soil characteristics and geomorphology, following Tomlinson *et al.* (2020). High-resolution spatial data for aspect, elevation and slope were sourced from Gallant and Austin (2012a) and Gallant and Austin (2012b), whilst spatial data describing the percentage of clay, silt and sand at 15-cm depth were sourced from Viscarra Rossel *et al.* (2014a), Viscarra Rossel *et al.* (2014b) and Viscarra Rossel *et al.* (2014c), respectively. These data were all aligned and downscaled to a consistent 1–arc-second resolution (~25 m^2^) by bilinear scaling using the elevation data as a template using the *‘raster’* package (Hijmans *et al.,* 2015) in the R statistical environment (R Core Team, 2021). Soil bulk density (milligramme/cubic metre) and depth were interpolated for each 25-m^2^ grid location from national soil data sourced from the Australian Collaborative Land Evaluation Program (ACLEP; www.clw.csiro.au/aclep).

We used the maximum entropy algorithm implemented in MaxEnt version 3.3.3a (Phillips *et al.,* 2006) to model the local distribution of *A. quadrata* in the three known populations along the southern edge of the Hamersley Range. Default MaxEnt parameter settings were used to develop logistic likelihoods of occurrence, with a value of 1 representing the highest likelihood (Phillips, 2008). To remove presence outliers, we applied a 10th percentile training presence, which excludes the 10% extreme (peripheral) observations. This was done to represent the ‘core’ of the known distribution and minimize the impact of uncharacteristic presence data.

We evaluated model performance by calculating the area under the threshold-independent receiver operating characteristic (ROC) curve (AUC), using values >0.9 to indicate well-validated models (Swets, 1988). We also calculated the True Skill Score (TSS) as a test of model robustness (Allouche *et al.,* 2006; Williams *et al.,* 2009) using the *evalSDM* function in the ‘mecofun’ v0.1.1 package (Zurell, 2020). Models with TSS <0.4 were identified as poor, whilst models with TSS >0.6 were identified as performing well (Beauregard and de Blois, 2014). We calculated a Boyce index of correlation between presence and suitability (Boyce *et al.,* 2002) using the *ecospat.boyce* function in the ‘ecospat’ package (Di Cola *et al.,* 2017), where values close to zero indicate models with predictive performance no better than random, and models close to 1 indicate strong predictive performance (Hirzel *et al.,* 2006). We also tested the significance of the partial response curves using *pROC* function in the ‘ntbox’ package (Osorio-Olvera *et al.,* 2020). These performance metrics were calculated over 100-iteration bootstraps using 10% test presence, which reserves 10% of the known occurrence locations for testing the resulting models (Phillips *et al.,* 2006; Phillips and Dudik, 2008). A full array of the test statistics available is presented in Supplementary Table 1.

Pilot models were developed using all the available candidate layers (elevation, aspect, slope, clay, sand and silt content and bulk density) and were further refined by removing layers that contributed <5% contribution to fit (Supplementary Fig. S2). The edaphic factors that the MaxEnt algorithm determined to be the best predictors of the probability of occurrence of *A. quadrata* were slope (percent), elevation (metres), soil bulk density (milligramme/cubic centimetre) and silt content (percent). As such, the final model was refined to these variables (Supplementary Fig. S2). The spatial projection was defined to encompass three IBRA bioregions (Thackway and Cresswell, 1997): the PIL, Little Sandy Desert (LSD) and GAS.

We interpolated a climate model to estimate microclimatic conditions associated with the spatial projection of the MaxEnt distribution model in line with the methodology and justification outlined in (Tomlinson *et al.,* 2020). Essentially, microclimatic projections for summer (wet) and winter (dry) ambient air temperature, surface soil temperatures, soil water potential at 20-cm depth and solar radiance were calculated and averaged using the ‘micro_global’ algorithm of the *‘NicheMapR’* statistical package (Kearney and Porter, 2017 in R (R Core Team, 2021). We downscaled our spatial data to 20–arc-second resolution (~300 km^2^), resulting in 1 651 622 grid point locations. At each point location, representing the centroid of the associated grid square, the physical soil characteristics were summarized into a format appropriate for *‘NicheMapR’* following a freely available soil texture calculator produced by the US Department of Agriculture (Soil Texture Calculator | NRCS Soils (usda.gov)) adapted to a computer algorithm similar to Gerakis and Baer (1999). For each point location we calculated hourly microclimatic conditions for every day of the year, using five replicate years’ resampling from the interpolated climate model (New *et al.,* 2002). Hourly values were then summarized to average daily conditions. For lack of any quantified proxies for vegetation shading, all microclimatic projections were run assuming full sun, with recognition that this does not capture all the microclimatic variation across the course of the day.

**Table 1:** Test statistics from one-way analysis of variation examining the microclimatic correlates of the likelihood of occurrence for *A. quadrata* based on microclimatic conditions calculated at 1000 random point samples across the projected landscape

**Season**	**Factor**	**F**_**(1,991)**_	**Pr (>F)**
Summer wet	Solar radiance (lumens)	0.968	0.325
	Ambient temperature (°C)	75.963	**<0.001**
	Soil temperature (°C)	0.782	0.377
	Soil water potential (kPa)	103.366	**<0.001**
Winter dry	Solar radiance (lumens)	21.079	**<0.001**
	Ambient temperature (°C)	0.034	0.854
	Soil temperature (°C)	16.729	**<0.001**
	Soil water potential (kPa)	135.026	**<0.001**

We identified four consecutive 90-day periods when air temperature was warmest, when air temperature was coldest and with the highest and lowest rainfall, respectively. At each location, hourly values were summarized as daily averages for these 90-day periods were again summarized to a mean wettest and driest quarter average for each point location over a 10-year period. In order to rescale these data back to the native 1–arc-second resolution, we used an interpolation approach (Carter *et al.,* 2018), where the microclimatic data at our 20–arc-second resolution were fed into a generalized linear model (GLM) informed by the edaphic and geomorphological data for each location. We generated unique GLMs for each microclimatic parameter for the wettest and driest quarters using the *‘stats’* package. We then used these GLMs to estimate the same parameters at point locations describing the grid centroids of the 1–arc-second landscape using the ‘predict’ function in R.

We extracted the climate data for 1000 random points within the training extent of the MaxEnt distribution model to construct a linear model describing the microclimatic correlates of the modelled likelihood of occurrence and habitat suitability. Following the construction of a ‘full’ model, we applied a model reduction using the ‘dredge’ function within the ‘*MuMIn’* package (Bartoń, 2014), and the models were examined by Akaike’s Information Criterion for small sample sizes (AICc; Burnham and Anderson, 2002). However, model reductions did not substantially increase model parsimony and the full model was retained and reported (Table 1).

### Microclimatic conditions in contrasting sites

To further evaluate soil microclimatic conditions between high- and low-probability sites, volumetric soil moisture content (cubic metre/cubic metre) and soil temperature (degrees Celsius) were measured in the field using HOBO® Micro Station Data Loggers (Onset Computer Corporation) that were fitted with two soil moisture (EC-5 ECH_2_O Dielectric Aquameter, Decagon Devices, Inc.) and two soil temperature probes (S-TMB-Temperature Smart Sensors, Onset Computer Corporation). The probes were buried at approximate depths of 300 mm of field and were set to log moisture content and temperature every 15 min for the entire duration of the study period (August 2021–October 2022). To convert volumetric moisture content to soil water potential, water retention curves were determined from soil composite subsamples extracted from each site, whereby three replicates of at least 5 g were saturated with water to obtain ‘field capacity’ moisture availability, followed by repeated oven drying at 75°C with soil moisture measurements undertaken every 10 min using a dew point psychrometer (WP4C Dew Point PotentiaMeter, Decagon Devices, Inc.) until the measured soil water potentials were drier than −100 MPa.

### Ecophysiological assessments

Physiological measurements in *A. quadrata* and *E. latrobei* were conducted over six monitoring periods (22–29 August 2021, 24–31 October 2021, 13–19 March 2022, 19–23 May 2022, 4–8 August and 13–18 October 2022) at two sites with contrasting modelled SDM probabilities. The average predicted probability of the high (23.180062°S, 117.423802°E) and low (23.180829°S, 117.427142°E) sites were 0.745 and 0.214, respectively, and were selected at similar landscape positions that were elevated and outside of major hydrological drainage areas, or creek lines. The average height × width of the measured plants was 139 ± 7 cm × 103 ± 10 cm and 152 ± 5 cm × 106 ± 5 cm for *A. quadrata* and *E. latrobei*, respectively. We did not find significant changes in plant sizes over the study period in plants.

### Gas exchange: photosynthetic rate, stomatal conductance and transpiration rate

For each of the species, photosynthetic rate (*Amax*) and stomatal conductance (*g_s_*) were measured using a LI-6400XT portable photosynthesis system and gas exchange analyser (LI-COR Biosciences, Lincoln, NE, USA) that was equipped with a 6400–40 leaf chamber fluorometer. All measurements were conducted between 0800 and 1200 pm, representing the time where the plant is most photosynthetically active prior to stomatal closure at solar noon. All measurements were quantified under constant light-saturated conditions, whereby photosynthetic active radiation was maintained at 1200 μmol m^−2^ s^−1^. Additionally, internal carbon dioxide concentrations were equilibrated to 400 μmol CO_2_ mol ^−1^ and relative humidity was maintained between 50 and 70%. Thermal conditions were maintained at ambient throughout all measurements to reflect seasonal temperature conditions at the time of measurement. All measurements were quantified on 10 replicate plants. On each plant, at least three replicate measurements were quantified on 2–3 individual tufts comprised of mature needle-like leaves that were located on the terminal stem. For each of the measurements, leaf tufts were allowed to equilibrate to the internal leaf chamber conditions, whereby the stability of gas exchange parameters was monitored in real time. Following measurement, leaf tufts that were measured were harvested from the plant and returned to the ecophysiology laboratory for leaf area analysis at Kings Park Science. All measurements were leaf-area corrected prior to statistical analysis.

### Leaf water potential

Leaf water potential measurements were conducted in order to determine plant available water (predawn measurements) and plant water status at the time of stomatal closure (midday measurements) (Turner, 1981). Predawn (*Ψ_pd_*) sampling occurred prior to first light (between 0300 and 0400 am), whereby terminal stems that were ~10 cm in length were harvested from plants and stored in a sealed foil bag in cool conditions, prior to leaf water potential assessment. Midday (*Ψ_md_*) sampling occurred approximately between 1045–1100 am during summer and between 1100–1200 pm in winter, representing the conditions of peak stress and approximate solar noon for the region. All measurements were conducted within 15–30 min of harvesting, whereby terminal stems were cut at a 45° angle and immediately secured within a Scholander Pressure Chamber (Model 1000, PMS Instruments Co, USA) with the cut stem externally exposed prior to pressurization (<100 bar). For each species, 10 replicate plants were measured, whereby 2–3 measurements were quantified per plant for *Ψ_pd_* measurements, and a single replicate measurement quantified per plant for *Ψ_md_* measurements.

*Chlorophyll performance: maximum quantum yield and electron transport rate.*

Prior to *Ψ_pd_* assessment, chlorophyll fluorescence measurements relating to maximum quantum yield (*F_v_/F_m_*) were quantified using a chlorophyll fluorometer (PocketPI, Hansatech Instruments Ltd, UK) on leaf tufts for each replicate terminal stem, resulting in 2–3 replicate measurements across 10 plants for each species, per site. Dark adaptation was not required for leaf tufts, as stems were harvested in the dark during the predawn measurement window. Electron transport rate (ETR) measures were conducted simultaneous to gas exchange measurements using the leaf fluorometer chamber attached to the LI-6400XT (see above, gas exchange measurements). For *ERT* measurements specifically, each of the three replicate tufts was measured a single time, equating to three measurements per plant, per site.

### Statistical analysis

Soil microclimate time series data (soil temperature and soil water potentials) at 30-cm depth were analysed used generalized additive models (GAMs) using the ‘gam’ function from the ‘mgcv’-package (Wood and Wood, 2015). For each microclimate variable, sites were considered a fixed effect to quantify microclimatic differences over the whole study period using a spline-based cubic regression smoothing term for each predictor, followed by an F-test with a global GAM without sites as a fixed effect. After fitting the GAM, the residuals of the spline-fit were visually inspected, then compared against different model combinations, smoothing terms and a linear model using AIC, R^2^ and RMSE (Wood and Wood, 2015; Haslbeck *et al.,* 2021) developed by the ‘compare_performance’ function in the ‘modelbased’-package (Makowski *et al.,* 2020).

All ecophysiological parameters (*A, g_s_, F_v_/F_m_, ETR, Ψ_pd_, Ψ_md_*) were analysed by fitting generalized linear mixed effects models (GLMMs), using ‘glmer’-function from the *‘lme4’*-package (Bates, 2010; Bates *et al.,* 2015) in the R statistical environment (R Core Team, 2021). For each ecophysiological parameter, we fixed species (*A. quadrata* and *E. latrobei*), site suitability (high and low) and the monitoring period (August 2021, October 2021, March 2022, May 2022, August 2022, October 2022) with *A. quadrata*, the high-suitability site and August 2021 determined as the model intercepts. For parameters (*Amax, gs, Ψ_pd_*) where we conducted multiple measurements across each plant, leaf replicate measurements were nested within plants for each monitoring period as the random effect. All main effects, as well all possible two-way and three-way interactions, were fitted, followed by assessing model strength via marginal and conditional R^2^ values (Schielzeth and Nakagawa, 2013). In addition, model assumptions (i.e. normality of residuals and random effects, linear relationship, homogeneity of variance and multicollinearity) for each ecophysiological parameter were assessed through graphical inspection with help of the ‘check_model’- function from the ‘performance’-package (Lüdecke *et al.,* 2021). When the data did not follow model assumptions, log- (for all parameters, except Fv/Fm) or logit- transformations were conducted, followed by refitting and visual inspection of the GLMM. Following model fitting, we performed type II Wald tests using the ‘Anova’-function in the ‘car’-package to evaluate fixed and interaction effects (Fox *et al.,* 2007).

## Results

### Species distribution modelling

**Figure 1:**
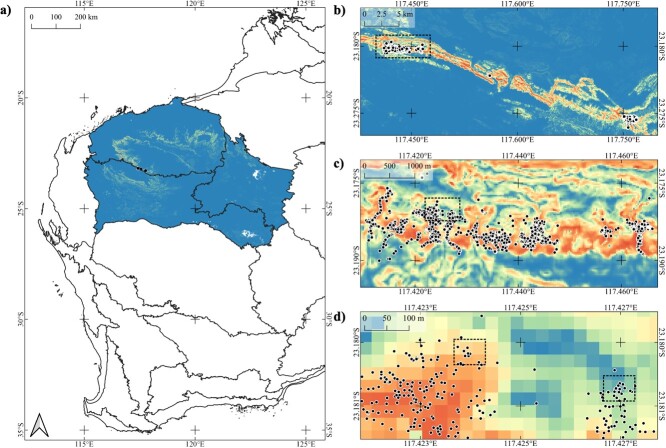
Map of the niche model indicating a) the distribution across three IBRA bioregions—PIL, LSD and GAS; b) Geographical extent of occurrence for known presences occurrences of *A. quadrata* defined by three distinct populations; c) the extent of the Western Ranges population; and d) the locations of the two study sites within the study zone. Increasing intensity of colour (from blue to red) indicates a higher probability of occurrence from 0 to 1 HSI.

The final species distribution model of *A. quadrata* was statistically robust, with high AUC (0.935; Pearce and Ferrier, 2000). The average habitat suitability index (HSI) at known occurrence locations was 0.68 (range = 0.02–0.92). Over 60% of the known occurrence locations (~27 700 individual plants) were modelled at habitat >0.7. Only 11% of individuals were modelled to occur in habitat with an HSI < 0.5. The strongest contributor to the modelled distribution was slope (56.2%) followed by elevation (13.1%) and bulk density (12.4%). High-suitability sites were associated with slopes of >15%, elevation between 425 and 445 m, an average soil bulk density of 1.41 g/cm^3^ and silt contents of <2%, whilst low-suitability sites were associated with slopes <10%, elevation between >460 and <420 m, soil bulk density greater than or <1.41 g/cm^3^ and silt contents of >2% (Supplementary Fig. S3). The northern fringes of the Hammersley Ranges were also predicted to have a high likelihood of occurrence, despite no known populations existing beyond the three populations identified along the southern extent of the range (Fig. 1). Additionally, the intervening area between the three extant populations is predicted to have a high likelihood (up to 98.2%) of supporting *A. quadrata.*

Microclimatic factors were significantly associated with habitat suitability of *A. quadrata* (adjusted R^2^ = 0.31, *F*_8,991_ = 56.37, *P <* 0.001). Model dredging identified the full linear model as the best description of the microclimatic effects (AICc = −1898.2, Log-likelihood = 959.232), revealing a positive relationship between habitat suitability and annual soil water potential (Summer Wet: *F*_1,991_ = 135.03, *P <* 0.001; Winter Dry: *F*_1,991_ = 103.37, *P <* 0.001; Table 1), followed by winter temperatures (*F*_1,991_ = 75.96, *P <* 0.001; Table 1), summer solar radiation (*F*_1,991_ = 21.08, *P <* 0.001; Table 1) and summer soil temperatures (*F*_1,991_ = 16.72, *P <* 0.001; Table 1).

### Soil microclimate variation

**Figure 2:**
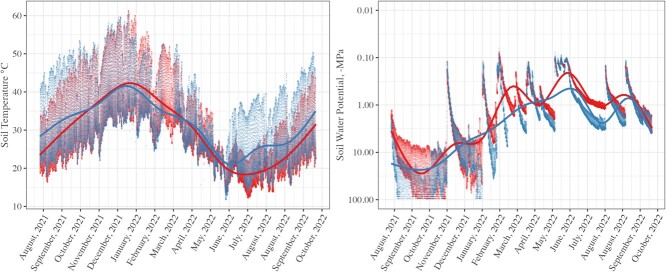
Soil microclimate variation for a) soil temperature and b) soil water potentials in high (red) and low (blue) suitability site. Microclimate parameters were measured *in situ* at 300-mm depth, recorded at 15-min intervals and were fitted with a spline curve to smooth the overall trends.

There were significant differences between *in situ* soil temperature (*t*-value = 36.69, *P* < 0.001, R^2^ = 0.530) and soil water potential (*t*-value = −57.04, *P* < 0.001, R^2^ = 0.576) between high- and low-suitability sites. On average, low-suitability sites were 0.58 times warmer and had 1.72 times drier conditions over the study period. The largest variation in temperatures for both sites was recorded during September 2021 to March 2022, coinciding with the periods leading up to summer rainfall, with minimum and maximum temperatures between 16 and 61°C (Fig. 2). During this period, median water potentials were ranging between −85.2 and −16.4 MPa in the low-suitability site and between −41.0 and −5.3 MPa in the high-suitability site. Thereafter, soils rehydrated following summer rainfall in both sites with median water potentials between −10.0 and −3.0 MPa in the low-suitability site and between −6.4 and −1.0 MPa in the high-suitability site for the months of January–March 2022 (Fig. 2). Late summer, autumn and winter rainfall events (between April and September 2022; Fig. 2.0 and Supplementary Fig. S3) further elevated median soil water potentials in both sites to between −1.0 and −0.2 MPa in the low-probability site and −0.9 and −0.2 MPa in the high-probability site.

### Ecophysiological assessment

**Figure 3:**
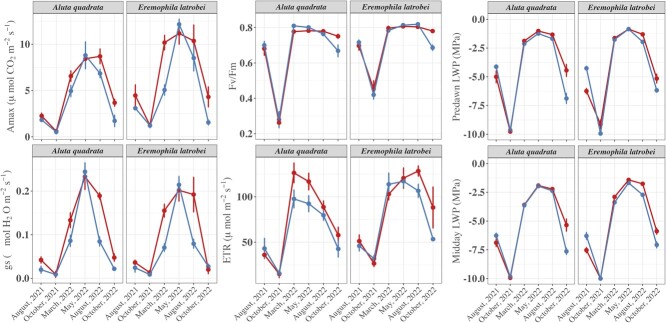
Seasonal variation in ecophysiological traits [photosynthetic rate (*Amax*), stomatal conductance (*g_s_*), maximum quantum yield (*Fv/Fm*), electron transfer rate (*ETR*), predawn and midday leaf water potential (LWP)] in *A. quadrata* and *E. latrobei* over six monitoring periods between August 2021 and October 2022, representing winter dry, presummer dry, and summer wet conditions in sites with high (red) and low (blue) habitat suitability, as determined through MaxEnt modelling. Point estimates are mean responses ± standard errors; *n* = 10 plants for each species

There were significant site-level differences between all ecophysiological parameters, except for *Fv/Fm* ratios (Table 2; all *P <* 0.029). Overall, plants in the high-suitability site had ecophysiological responses of up to 24% greater magnitude compared to those from the low-suitability sites (Fig. 3). The most responsive parameters were associated with gas exchange (*Amax*: X^2^ = 15.10, *gs*: X^2^ = 56.74; both *P* < 0.001), driven by site differences in March, August and October 2022 (Fig. 3; all *P* < 0.001). Species-level differences were characterized by *E. latrobei* having a higher photosynthetic rate, *Fv/Fm, ETR* and predawn leaf water potentials (Fig. 3; all *P <* 0.011), but not for stomatal conductance and midday leaf water potentials (Table 2).

**Table 2:** Summary statistics for ecophysiological responses from GLMMs for *E. latrobei* in high-suitability (intercept) and low-suitability sites, and the monitoring period (August 2021 (intercept), October 2021, March 2022, May 2022, August 2022 and October 2022)

		**A**		**gs**		**Fv/Fm**		**ETR**		**PD**		**MD**	
*Model terms*	*df*	*X^2^*	*P*	*X^2^*	*P*	*X^2^*	*P*	*X^2^*	*P*	*X^2^*	*P*	*X^2^*	*P*
Site suitability (S)	1	**15.10**	**<0.001**	**56.74**	**<0.001**	1.59	0.21	**6.72**	**<0.01**	**6.10**	**0.013**	**4.72**	**0.029**
Species (Sp)	1	**30.43**	**<0.001**	1.16	0.281	**90.17**	**<0.001**	**19.39**	**<0.001**	**6.53**	**0.011**	0.57	0.448
Month (M)	5	**16.41**	**<0.01**	**13.07**	**0.023**	**30.97**	**<0.001**	409.43	**<0.001**	**135.25**	**<0.001**	**67.95**	**<0.001**
S × M	5	10.28	0.068	**47.35**	**<0.001**	**24.53**	**<0.001**	6.48	0.261	**200.17**	**<0.001**	**30.68**	**<0.001**
S × Sp	1	**5.94**	**0.014**	0.97	0.324	**5.70**	**0.017**	0.94	0.332	0.51	0.47	0.32	0.566
Sp × M	5	8.34	0.138	1.69	0.889	**133.82**	**<0.001**	**12.21**	**0.032**	**29.96**	**<0.001**	7.45	0.188
Sp × S × M	5	2.34	0.674	3.77	0.438	**9.75**	**0.045**	8.14	0.148	**17.07**	**<0.01**	11.01	0.051
													
Marginal R^2^/conditional R^2^	0.439/0.655			0.506/0.730		0.717/0.888		0.265/0.843		0.896/0.939		0.823/0.901	

There was strong seasonal variation in plant performance in both species between August 2021 and October 2022 as indicated by all ecophysiological parameters (Table 2 and Fig. 3). Gas exchange rates varied by >5 mmol CO_2_ m^−2^ s^−1^, with stomatal conductance also varying by >0.075 mol H_2_O m^−2^ s^−1^ over this time (Fig. 3). Variation in other parameters was observed: chlorophyll-based measures *F_v_/F_m_* > 0.65 and ETR responses >80 mmol electrons m^−2^ s^−1^; and *Ψ_pd_* as well as *Ψ_md_* leaf water potential measurements varying between −1.2 and −2.4 MPa and −1.8 and −3.3 MPa, respectively (Fig. 3). By contrast, measurements in August and October 2021 represented 50–95% in reductions from the maximum responses between March and May 2022 in ecophysiological performance. Gas exchange rates were <2 mmol CO_2_ m^−2^ s^−1^, and stomatal conductance was <0.025 mol H_2_O m^−2^ s^−1^ in both species (Fig. 3). Chlorophyll performance was reduced to maximum quantum yield measures of *F_v_/F_m_* < 0.3 and decreased *ETR* responses <50 μmol electrons m^−2^ s^−1^ (Fig. 3). As well, traits associated with plant water stress indicated low plant available water, with *Ψ_pd_* as well as *Ψ_md_* < −8 MPa (Fig. 3).

## Discussion

By integrating a high-resolution SDM with mechanistic measurements of seasonal variation in ecophysiological performance, we have demonstrated a strong association between modelled habitat suitability in the SRE, *A. quadrata* with ecophysiological performance. Plants growing at sites with high modelled suitability according to remotely sensed edaphic and geomorphological conditions had higher rates of ecophysiological performance across most of the traits that we measured. We posit this as validation that modelled likelihood of occurrence is indicative of habitat suitability for *A. quadrata*. In comparison with *E. latrobei*, we found that the SRE *A. quadrata* had decreased photosynthetic activity, chlorophyll fluorescence and predawn leaf water potentials, indicating species-level differences, even when measured in the same environment. The knowledge generated from this study will help to better understand *A. quadrata* within its environment and lead to improved management and conservation of this species and potentially other SRE species more broadly.

### Patterns of modelled habitat suitability

By modelling the distribution, we identified locations varying in probability of occurrence based on the correlation of occurrence data with edaphic factors. Here, *A. quadrata* was modelled to occur predominantly on elevated, rocky slopes along the Hamersley Ranges. Of particular note, our SDM projected only 0.1% of the potential *A. quadrata* distribution with likelihood of occurrence, inferred as habitat suitability (Guisan *et al.,* 2017) >0.7. The modelled preference of *A. quadrata* for elevated, mesic habitats, with high slope percentages and shallow, well-drained soils with low silt contents are characteristic of SRE species persisting on similar geological land forms (Gibson *et al.,* 2012; Di Virgilio *et al.,* 2018; Robinson *et al.,* 2019; Tomlinson *et al.,* 2020). Also, consistent with modelling distributions of SRE species in similar geological land forms (Tomlinson *et al.,* 2020), the SDM identified substantial areas of high suitability throughout the Pilbara (~1132 km^2^), which is outside the known extent of *A. quadrata*. The modelling approach of this study is valuable in identifying these pockets of suitable habitat, both in proposing likely locations of unidentified populations of the species (White *et al.,* 2020) or for guiding translocations (Guisan *et al.,* 2017; Draper *et al.,* 2019), especially where vacancy of such habitats is typically ascribed to stochastic extinction or failure of the species to disperse there naturally (Byrne *et al.,* 2019).

Although modelled likelihood of occurrence is often assumed to indicate habitat suitability (Gogol-Prokurat, 2011; Guisan *et al.,* 2017), a common challenge for SDM projections is to extrapolate from this inferred suitability to verified species performance (Hereford *et al.,* 2017). We found that inferred habitat suitability was strongly associated with differences in ecophysiological performance of *A. quadrata*, such that individuals in high-suitability sites had higher physiological performance compared to the low-suitability site. In addition to the spatial contrasts explored here, we found temporal variation as seasons transitioned between peak functioning after rainfall and stress periods between seasons (e.g. March–August 2022 and October 2022, respectively) and years (August and October 2021 and 2022). These patterns demonstrated clear climate-driven underpinnings to habitat suitability at these sites, with plant activity and inactivity found in response to rainfall and drought, respectively. Nevertheless, differences in plant performance between these sites disappeared during seasons of increased water stress (e.g. August and October 2021), indicating that high average suitability does not preclude a site from imposing substantial challenges to the local population during high-stress periods, and rather, climatically favourable seasons drive site differences. As such, although habitat suitability modelling trained using edaphic traits can provide more accurate projections at finer resolution than those trained on climatic data (Tomlinson *et al.,* 2020), the greatest challenge to such modelling approaches is to infer climatic patterns and to project these to estimate the effect of changing climates and the increased likelihood of extreme climatic events on SRE plant populations.

Our approach was advocated for on the basis of the standardized training layers allowing for directly comparable models to be developed for similar species anywhere in Australia (Tomlinson *et al.,* 2020). The 25-m^2^ spatial resolution does, however, lead to smoothing or averaging of microtopographic variation of edaphic factors within each modelled grid cell. Other studies modelling the distribution of SRE plants have used LiDAR technology to map microtopographic features at a 2-m resolution (Di Virgilio *et al.,* 2018; Robinson *et al.,* 2019), and downscaling may further help understand landscape variation at a local scale. The advantage to the edaphic layers that we used here is that they can be directly fed into biophysical models to downscale microclimatic conditions at each site (Kearney and Porter, 2009; Tomlinson *et al.,* 2020). Here, we found that these microclimatic inferences closely correlated with plant physiological traits, which could theoretically be used to project likely performance under modelled future climates. Nevertheless, such projections are always going to represent inferences made on the basis of statistical correlations, and recent studies have employed mechanistic models informed by phenophysiological responses of species in relation to microclimatic niche gradients (Hereford *et al.,* 2017; Schouten *et al.,* 2020). These models can be particularly insightful, as they have the capacity to simulate environmental stressors across a plant life cycle (Schouten *et al.,* 2020), potentially identifying critical stages that govern population growth, reproduction and persistence. By scaling these models across the distribution, at large management scales, it is theoretically possible to determine management triggers based on projected plant performance, but the nature of mechanistic models is to overestimate the realized niche by identifying climatically suitable space without reference to biotic filters (Peterson *et al.,* 2015). A hurdled modelling approach (Ridout *et al.,* 1998) may optimize the predictive potential of both techniques, where edaphically informed SDMs are used identify a template of suitable habitats, and then mechanistic models are applied within this constrained space to estimate plant performance under changing conditions.

### *Ecophysiology of* A. quadrata *and comparisons with* E. latrobei

Generally, the ecophysiological performance of *A. quadrata* correlated well with modelled habitat suitability. Nevertheless, there were seasonal patterns in plant performance that were not well represented in the modelling, especially in seasons of extreme physiological stress. As seasons transition from the wet into the dry (e.g. during October 2022), ecophysiological activity was characterized by downregulation of gas exchange and reductions in chlorophyll fluorescence, indicating changes from productive growth phases during the wet season to plant senescence in the dry season (Manzoni *et al.,* 2011; Vico *et al.,* 2015). Whilst these patterns present typical responses of plants to shifts in seasonal water availability (Chaves *et al.,* 2003; Ogle and Reynolds, 2004), both species persisted through intense plant water stress conditions. For example, during the period of highest water stress (lowest measured water availability), when predawn leaf water potentials were −9.1 to −9.9 MPa and soil water potentials were <−2 MPa stress (e.g. October 2021), we found up to 65% reductions in chlorophyll fluorescence metrics (*Fv/Fm* < 0.3 and *ETR* < 50 μmol electrons m^−2^ s^−1^) and up to 95% in reductions in photosynthetic activity and stomatal conductance in both species. Whilst for many plant species optimal ranges for *Fv/Fm* ratios typically vary between 0.75 and 0.83 (Maxwell and Johnson, 2000; Schönbeck *et al.,* 2023), a reduction of *Fv/Fm* ratios <50% efficiency is typically associated with very low plant health and an increased likelihood of mortality due to photoinhibition (Demmig-Adams and Adams III, 2006). In addition, previous research has reported that recovery of photosynthetic activity in several species is not possible if stomatal conductance responses are lower than the severe drought threshold of 0.05 mol H_2_O m^−2^ s^−1^ (Flexas *et al.,* 2006). However, for both species, we did not observe plant mortality in any of the individuals over the study period, with plants recovering to *Fv/Fm* ratios >0.75 in the wet season. Therefore, in highly seasonal landscapes like the PIL and GAS region, the biogeographical filters that lead to short-range endemism may be dependent on seasonal or ephemeral conditions.

Interestingly, whilst gas exchange measures presented site-level differences, *Fv/Fm* measures did not demonstrate the same level of variation. This could be explained by *Fv/Fm* measures representing the maximum potential efficiency of the photosystem II (PSII), which is a result of environmental variation in stressors during the seasonal window impacting on physiological activity and morphological adjustments to leaves (Maxwell and Johnson, 2000), rather than the instantaneous changes in the environment impacting on photosynthetic activity. In addition, the edaphic characteristics of the contrasting sites may have been pronounced at the same level of plant water stress, but not to the extent to cause severe impairment in PSII. By contrast, *ETR* responses demonstrated stronger variation than *Fv/Fm* ratios, which is likely explained by this trait more strongly correlating with photosynthetic activity rates (Galmés *et al.,* 2007). Nevertheless, the pattern of recovery from the dry October 2021 to the wet March 2022 confers adaption for both species to their water-limited environment and the ability to withstand periods of severe drought stress.

Our study found species-level differences in physiological activity that were characterized by elevated photosynthetic activity in *E. latrobei* in contrast to *A. quadrata*, whilst presenting similar stomatal conductance responses over seasons and across sites. These responses typically indicate higher intrinsic water use efficiency (WUEi; the ratio between photosynthetic activity and stomatal conductance, *A/gs;* see Fig. 3 and Supplementary Fig. S5) which likely presents increased water stress tolerance for *E. latrobei* (Atkin *et al.,* 1998; Kimball *et al.,* 2016; Valliere *et al.,* 2021). In addition, higher WUEi and photosynthetic rates may also suggest increased growth rates and a competitive advantage for resources over *A. quadrata* in the same environment (Tezara *et al.*, 2010; Tarin *et al.,* 2020). Despite the differences in WUEi, both species displayed average decreases of up to −2.14 MPa in midday leaf water potentials relative to their predawn measures, as well as decreasing stomatal conductance at moderate to high leaf water potentials as seasons transition. At this scale, these responses suggest both species to be anisohydric, which maintain higher stomatal conductance rates in contrast to isohydric species, allowing for leaf water potentials to decline with decreasing soil water potential (McDowell *et al.,* 2008). Whilst our climate data showcase that plants can persist for at least 103 days between August and November 2021 without any rainfall (see Supplementary Fig. S3), and in a landscape that was beginning to experience thermal extremes as seasons were transitioning into the hot summer period, there is uncertainty about how long both species could continue to survive in a period of longer term drought. Their ability to recover without mortality over such period further supports that both species are highly adapted to their arid environment. However, further investigation under controlled environmental studies or field surveying is necessary to understand their drought survival capacity and threshold for mortality over sustained periods of severe water deficit. Additionally, whilst our study was focused on reproductive, adult plants, it is likely that tolerance to seasonal stressors would vary between seedling, juvenile and adult states, and further research is necessary to identify ontogenetic sensitivities to abiotic stress (Lewandrowski *et al.,* 2021; Gremer, 2023). These physiological data can work to optimize emerging mechanistic models (Schouten *et al.,* 2020) and increase capacity to explain and predict changing spatiotemporal patterns or population dynamics to guide conservation action for SRE plants.

### Conservation implications

Recent studies have emphasized the importance of understanding biogeographical (Draper *et al.,* 2019) as well as ecophysiological (Madliger *et al.,* 2018; Cooke *et al.,* 2021) contexts of species for conservation. When combined, these approaches can provide strategic applications for plant conservation and ecological restoration (Madliger *et al.,* 2018; Tomlinson *et al.,* 2021; Valliere *et al.,* 2021; Schönbeck *et al.,* 2023). Given high physiological activity is associated with increased productivity and reproductive success of individuals, highly suitable locations where the species are present should be considered for targeted conservation of the species. Research rarely intensively ground-validates model predictions, but where this has been done, high-suitability habitats have been found to harbour previously identified populations of SRE species (White *et al.,* 2020). Nevertheless, habitat with high modelled suitability can also be used as recipient locations for conservation translocations (Draper *et al.,* 2019), given the high risk of stochastic losses of short-range endemic plant populations (Bartholomeus *et al.,* 2011).

The modelled habitat suitabilities that we identified have proven highly correlated with physiological traits governing species persistence. However, from an applied perspective, such spatiotemporal variation can lead to a high level of uncertainty, especially when ecophysiological measurements used to validate SDM outputs are conducted in a dry season, highlighting the importance for undertaking contrasting seasonal measurements in climatically stochastic landscapes (Grossman, 2023). Nevertheless, whilst our study only investigated spatiotemporal variation in contrasting sites, the next logical step for research is to account for greater variation in landscape ecotypes and maximize spatial variability. Many *A. quadrata* plants are distributed along drainage channels in varying degrees of slope angles, elevation and soil bulk densities, which could further impact physiological activity. By evaluating the interactions of these edaphic factors, we will likely increase our understanding of the patterns and processes underpinning plant performance across the landscape, and deliver evidence-based insight into the ongoing management and conservation of the threatened SRE.

## Supplementary Material

Web_Material_coae030
