# Supplementary material for: Spatiotemporal variation in ecophysiological traits align with high resolution niche modelling in the short-range banded ironstone endemic Aluta quadrata
Source: Conserv Physiol. 2024 May 24;12(1):coae030. doi: 10.1093/conphys/coae030 (PMC11127796; doi:10.1093/conphys/coae030)
Supplement: Web_Material_coae030 [file web_material_coae030.zip › Lewandrowski et al Aluta ms Supplementary materials_Second_submission.pdf]

Supplementary materials: Spatiotemporal variation in ecophysiological traits align with high resolution niche modelling in the short-range banded ironstone endemic *Aluta quadrata*

WOLFGANG LEWANDROWSKI[1,2*^], EMILY P TUDOR[1,2*], HAYDEN AJDUK[3], SEAN TOMLINSON[1,4,5], JASON C STEVENS[1]

[1]Kings Park Science, Department of Biodiversity, Conservation and Attractions, Kings Park, WA 6005, Australia,

[2]School of Biological Sciences, University of Western Australia, Nedlands, WA 6009, Australia

[3]Rio Tinto Iron Ore, GPO Box A42, Perth, WA 6000, Australia

[4]Geospatial Science, Department of Biodiversity, Conservation and Attractions, Kensington, WA 6151, Australia,

[2]School of Biological Sciences, University of Adelaide, Adelaide, SA 5000, Australia

*shared first author, both authors have contributed equally to the manuscript

^corresponding author: wolfgang.lewandrowski@dbca.wa.gov.au

Items

Supplementary Figure S1: Known occurrence locations of the short-range endemic *Aluta quadrata* and the more widespread *Eremophila latrobei* across north-west Western Australia. The filled area encompasses the Pilbara (PIL), Little Sandy Desert (LSD) and Gascoyne (GAS) IBRA regions, shaded by elevation from low elevation (dark) to high elevation (pale).

Supplementary Figure S2: A) Relative contributions of topographic and edaphic variables contributing to the *Aluta quadrata* MaxEnt species distribution model. B) Density of the four strongest contributors to low suitability (blue) and high suitability (red) habitat. C) spatial representations of the top four contributing microclimatic factors

Supplementary Figure S3: Microclimatic conditions throughout the study period. a) soil temperature and b) soil water potentials measured at 300mm depth, recorded every 15mins.

Two sites are presented in each panel, high suitability (red) and low (blue) suitability site. The data were fitted with a spline curve to smooth the overall trends for temperature and moisture measurements. Black columns represent monthly precipitation recorded at the nearest SILO climate location for the station point -23.20, 117.45 in close proximity to our study site (SILO climate database; https://www.longpaddock.qld.gov.au/silo/). The survey period falls within the vertical dashed red lines.

Supplementary Figure S4: Soil microclimate variation for a) soil temperature and b) soil water potentials in high (red) and low (blue) suitability site. Microclimate parameters outlined in black were measured *in situ* at 300mm depth, recorded at 15-minute intervals. Raw data are presented in Figure S3. Parameters outlined in colour were modelled for each site using the 'micro_global' algorithm in NicheMapR (Kearney, 2016).

Supplementary Figure S5: Partial response plots for the contributions to *Aluta quadrata* MaxEnt species distribution model.

Supplementary Figure S6: Intrinsic water use efficiency (WUEi, the relationship of photosynthetic rate (*Amax*) and stomatal conductance (*gs*) for *Aluta quadrata* and *Eremophila latrobei*. Point-estimates represent individual plant measurements (n=10) measured over different surveying months between August, 2021 and October, 2022. The higher photosynthetic rate at the same level of stomatal conductance in *E. latrobei* indicates higher WUEi in comparison to *A. quadrata*.

Supplementary Table 1: Model evaluation scores developed to test the robustness of our SDM of *Aluta quadrata*.

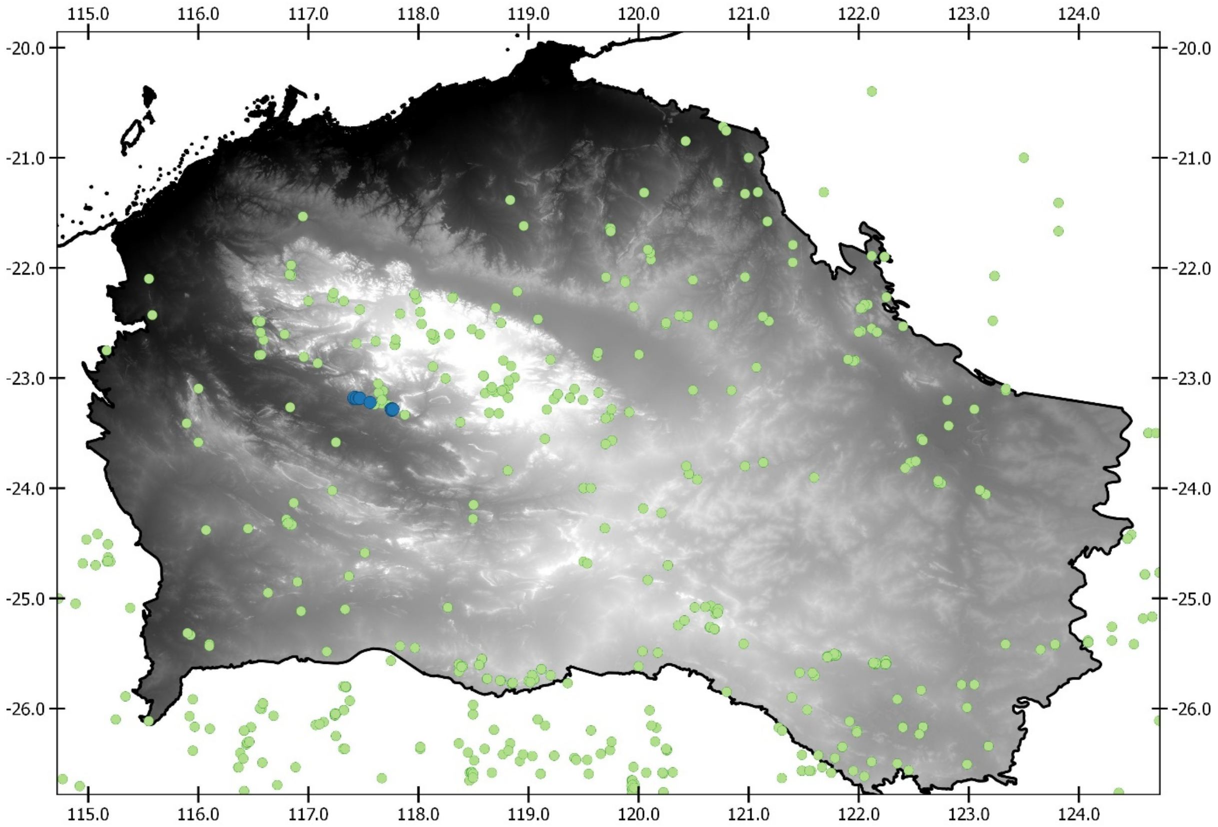

Supplementary Figure S1: Known occurrence locations of the short-range endemic *Aluta quadrata* and the more widespread *Eremophila latrobei* subsp. *glabra* (L.S.Sm.) Chinnock across north-west Western Australia. The filled area encompasses the Pilbara (PIL), Little Sandy Desert (LSD) and Gascoyne (GAS) IBRA regions, shaded by elevation from low elevation (dark) to high elevation (pale).

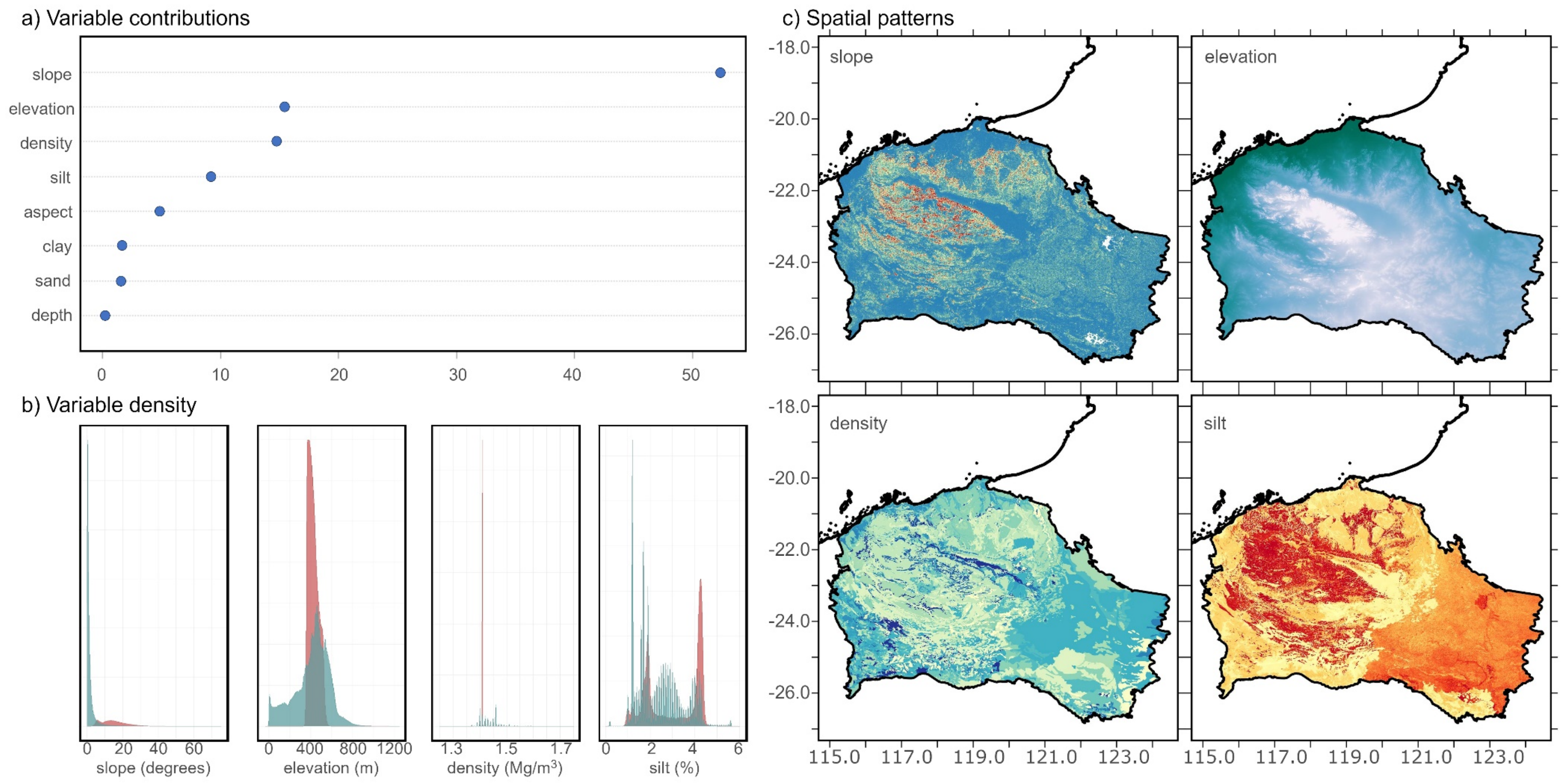

Supplementary Figure S2: A) Relative contributions of topographic and edaphic variables contributing to the *Aluta quadrata* MaxEnt species distribution model. B) Density of the four strongest contributors to low suitability (blue) and high suitability (red) habitat. C) spatial representations of the top four contributing microclimatic factors.

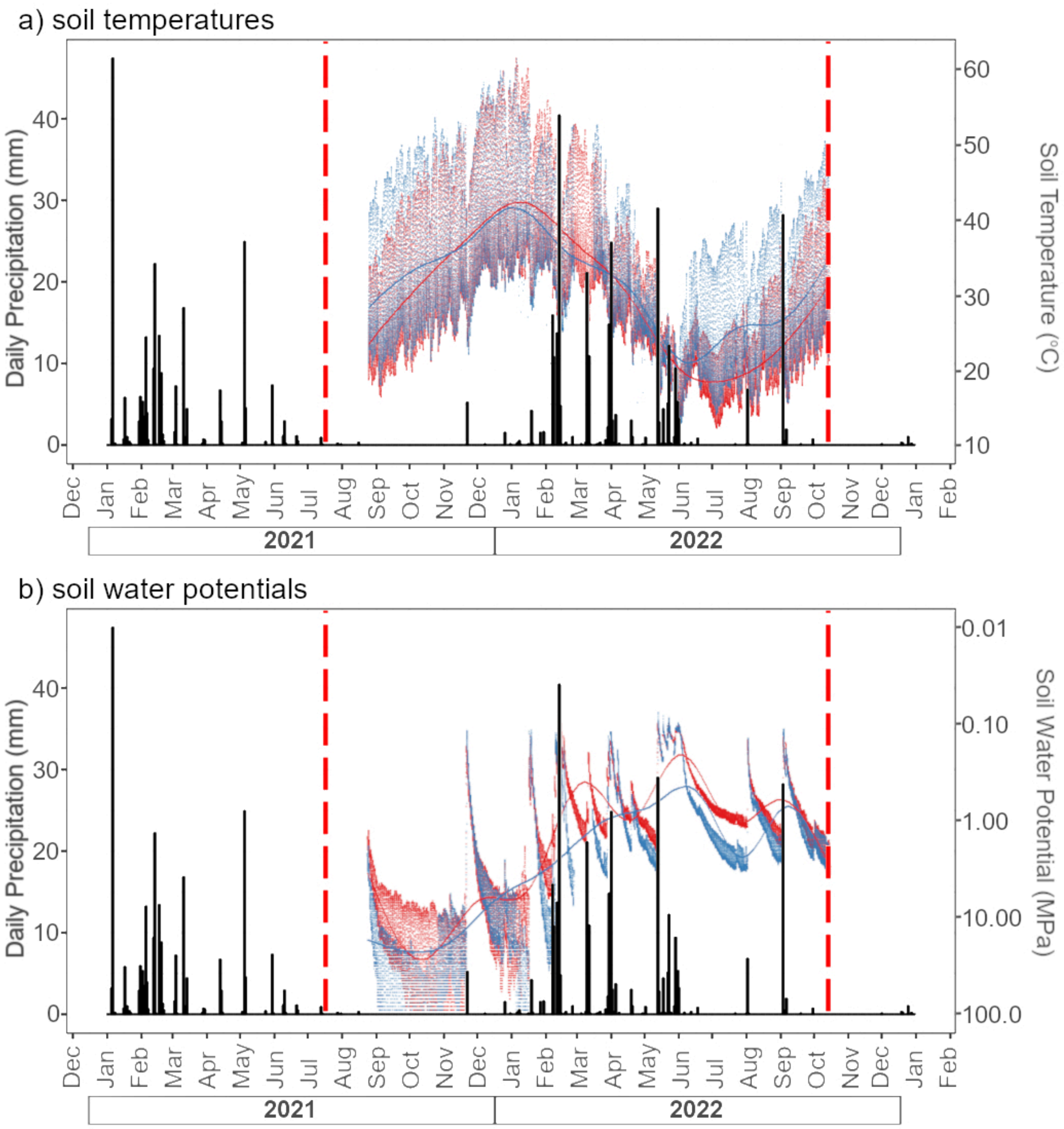

Supplementary Figure S3: Microclimatic conditions throughout the study period. a) soil temperature and b) soil water potentials measured at 300mm depth, recorded every 15mins. Two sites are presented in each panel, high suitability (red) and low (blue) suitability site. The data were fitted with a spline curve to smooth the overall trends for temperature and moisture measurements. Black columns represent monthly precipitation recorded at the nearest SILO climate location for the station point -23.20, 117.45 in close proximity to our study site (SILO climate database; https://www.longpaddock.qld.gov.au/silo/). The survey period falls within the vertical dashed red lines.

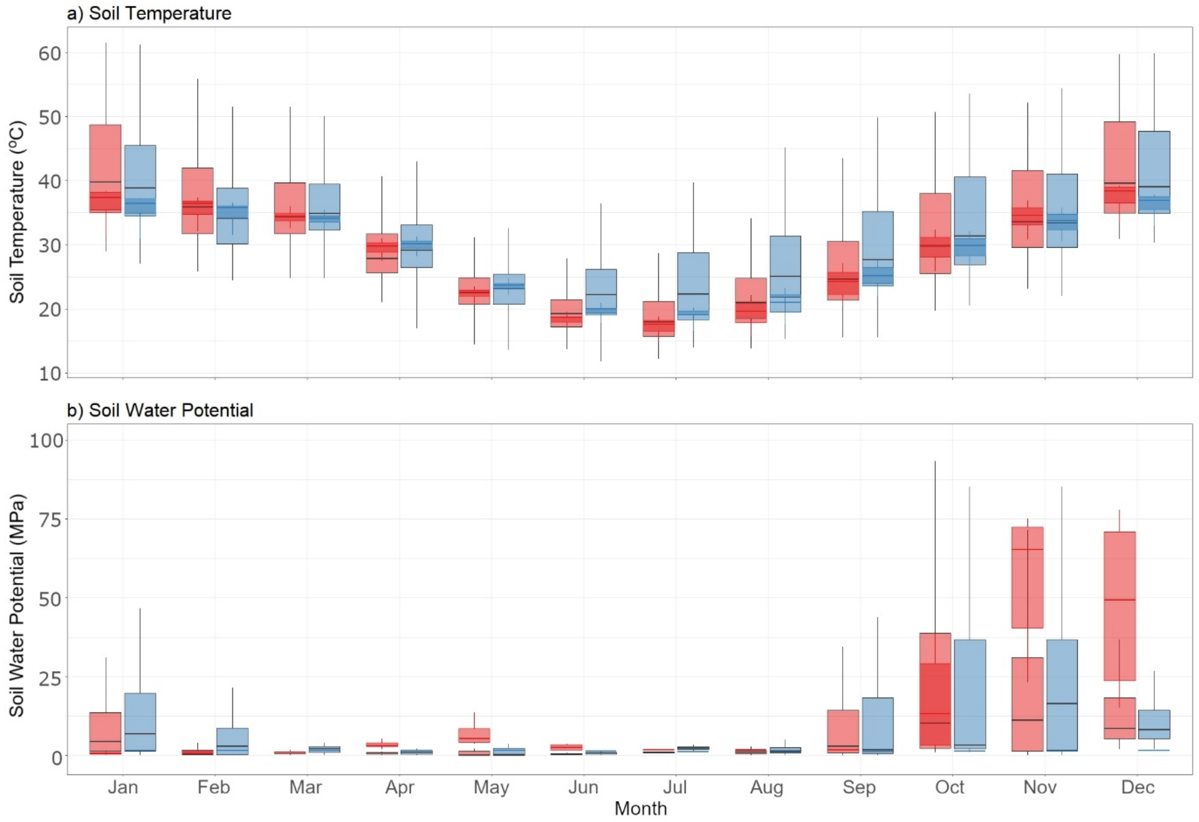

Supplementary Figure S4: Soil microclimate variation for a) soil temperature and b) soil water potentials in high (red) and low (blue) suitability site. Microclimate parameters outlined in black were measured *in situ* at 300mm depth, recorded at 15-minute intervals. Raw data are presented in Figure S3. Parameters outlined in colour were modelled for each site using the 'micro_global' algorithm in NicheMapR (Kearney, 2016).

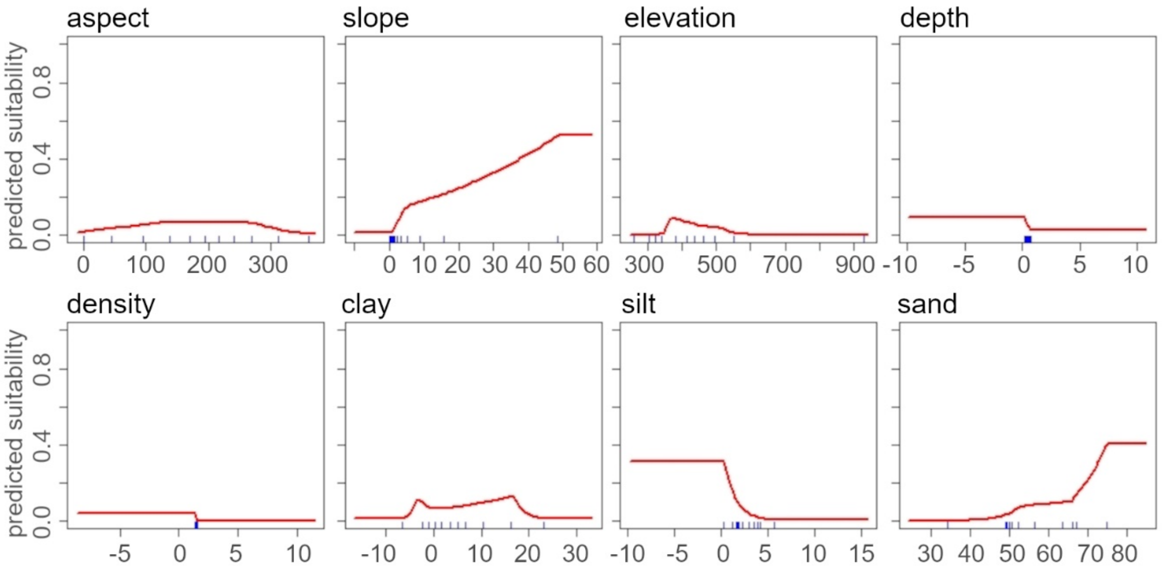

Supplementary Figure S5: Partial response plots for the contributions to *Aluta quadrata* MaxEnt species distribution model.

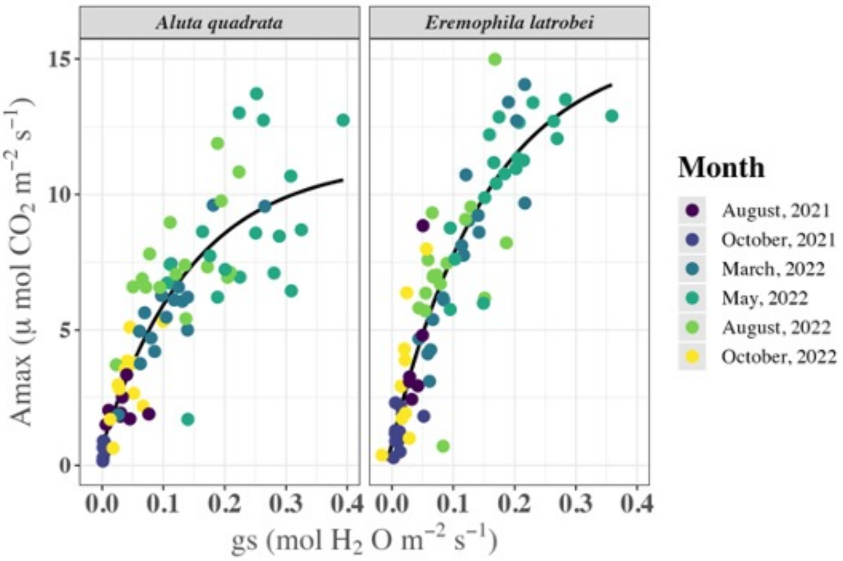

Supplementary Figure S6: Intrinsic water use efficiency (WUEi, the relationship of photosynthetic rate (*Amax*) and stomatal conductance (*gs*) for *Aluta quadrata* and *Eremophila latrobei*. Point-estimates represent individual plant measurements (n=10) measured over different surveying months between August, 2021 and October, 2022. The higher photosynthetic rate at the same level of stomatal conductance in *E. latrobei* indicates higher WUEi in comparison to *A. quadrata*.

Supplementary Table 1: model evaluation scores developed to test the robustness of our SDM of *Aluta quadrata*.

Metric	Score	p	Source	
AUC	0.988	-	*evalSDM*	(Zurell 2020)
TSS	0.912	-	*evalSDM*	(Zurell 2020)
Kappa	0.448	-	*evalSDM*	(Zurell 2020)
Sensitivity (true positives)	0.981	-	*evalSDM*	(Zurell 2020)
Specificity (true negative)	0.931	-	*evalSDM*	(Zurell 2020)
Proportion correctly classified (PCC)	0.933	-	*evalSDM*	(Zurell 2020)
Deviance explained (D2)	0.453	-	*evalSDM*	(Zurell 2020)
Binary threshold	0.220	-	*evalSDM*	(Zurell 2020)
Boyce	0.993	-	*ecospat.boyce*	(Di Cola et al. 2017)
pROC	1.885	<0.01	*pROC*	(Osorio-Olvera et al. 2020)
